# Coblation Adenoidectomy Versus Conventional Adenoidectomy: A Comparative Study of two Different Techniques of Adenoidectomy

**DOI:** 10.22038/ijorl.2025.84811.3855

**Published:** 2025

**Authors:** Dianitta-Devapriya Veronica, Prabaakharan Jambunathan

**Affiliations:** 1 *Department of Otorhinolaryngology and Head and Neck Surgery, ACS Medical College Hospital, Dr. MGR Educational and Research Institute, Chennai-India.*

**Keywords:** Adenoidectomy, Radiofrequency ablation, Endoscopy, Curettage, Postoperative pain

## Abstract

**Introduction:**

Chronic nasal obstruction, frequent respiratory infections, recurrent ear blocks, earaches, and pediatric obstructive sleep apnea may indicate adenoid enlargement, one of the most common conditions encountered in pediatric otorhinolaryngology practice. Adenoidectomy is a simple procedure with certain limitations, which has led to various innovations in surgical techniques in the recent past. The study aimed to compare two different adenoidectomy techniques: the endoscopy-assisted coblation adenoidectomy and the conventional curettage adenoidectomy.

**Materials and Methods:**

In this prospective randomized interventional study involving 40 patients, 20 patients in Group A underwent curettage adenoidectomy, and 20 patients in Group B underwent endoscopic coblation adenoidectomy. Complete adenoid tissue removal, surgical blood loss, operative duration, postoperative pain, and recovery time are the outcome measures.

**Results:**

Endoscopy-assisted coblation adenoidectomy enabled complete adenoid removal better than conventional adenoidectomy, 15 patients (75%) had complete removal versus 3 patients (15%) in the conventional group (p-value of 0.0003). The mean blood loss was 30 ± 5.60 mL in Group A and 10.75 ± 2.93 mL in Group B (p = 0.0001). The pain score assessed using the visual analog scale was 4 ± 0.44 in Group A and 3 ± 0.36 in Group B (p = 0.0001). The mean time taken for recovery in Group A was 3.14 ± 0.62 days and that in Group B was 2.64 ± 0.64 days (p = 0.001).

**Conclusions:**

Coblation adenoidectomy under endoscopic guidance enabled complete adenoid removal, reduction in surgical blood loss and postoperative pain, and shortened recovery time.

## Introduction

Symptoms such as persistent nasal obstruction, mouth breathing, snoring, and frequent ear blocks in pediatric patients may indicate adenoid enlargement. Chronic adenoiditis can lead to Eustachian tube dysfunction, resulting in otitis media with effusion. Additionally, chronic adenoiditis can act as a focal point for infections, contributing to recurrent respiratory issues and other dermatological conditions. These problems can lead to poor appetite, malnutrition, and growth retardation, which in turn can affect a child's concentration and school performance. Children with enlarged adenoids usually present with characteristic adenoid facies. In addition, high-grade adenoid hypertrophy can cause obstructive sleep apnea and eventually result in cor pulmonale (1). 

While adenoid enlargement is physiological, children with airway compromise or issues with facial skeleton development require it to be addressed surgically. Adenoid hypertrophy can be effectively treated with intranasal corticosteroids (2). However, surgery should be considered when medical treatments are unsuccessful. Simple curettage adenoidectomy has been a longstanding procedure, first pioneered by Hans Wilhelm Meyer in the 19th century, and has evolved significantly over the last century (3). 

The widespread use of endoscopes in ENT surgeries has led to coblation adenoidectomy under endoscopic guidance in recent days. Controlled ablation is the principle by which coblation technology works and the tissue volume is reduced by cellular disintegration at the molecular level (4). In contrast, conventional adenoidectomy is a blind procedure that can accidentally injure adjacent structures and may leave behind residual adenoid tissue, which can lead to recurrence (5). The endoscopic approach can mitigate this risk by enabling the clear visualization of adjacent structures, thereby minimizing the risk of injury during the complete removal of the adenoid. Endoscopy-assisted coblation technique is superior because it avoids tissue explosion; instead, it breaks down tissues at the molecular level into simpler hydrocarbons. The study aimed to compare endoscopy-assisted coblation adenoidectomy with conventional curettage adenoidectomy.

## Materials and Methods

**Fig 1:**
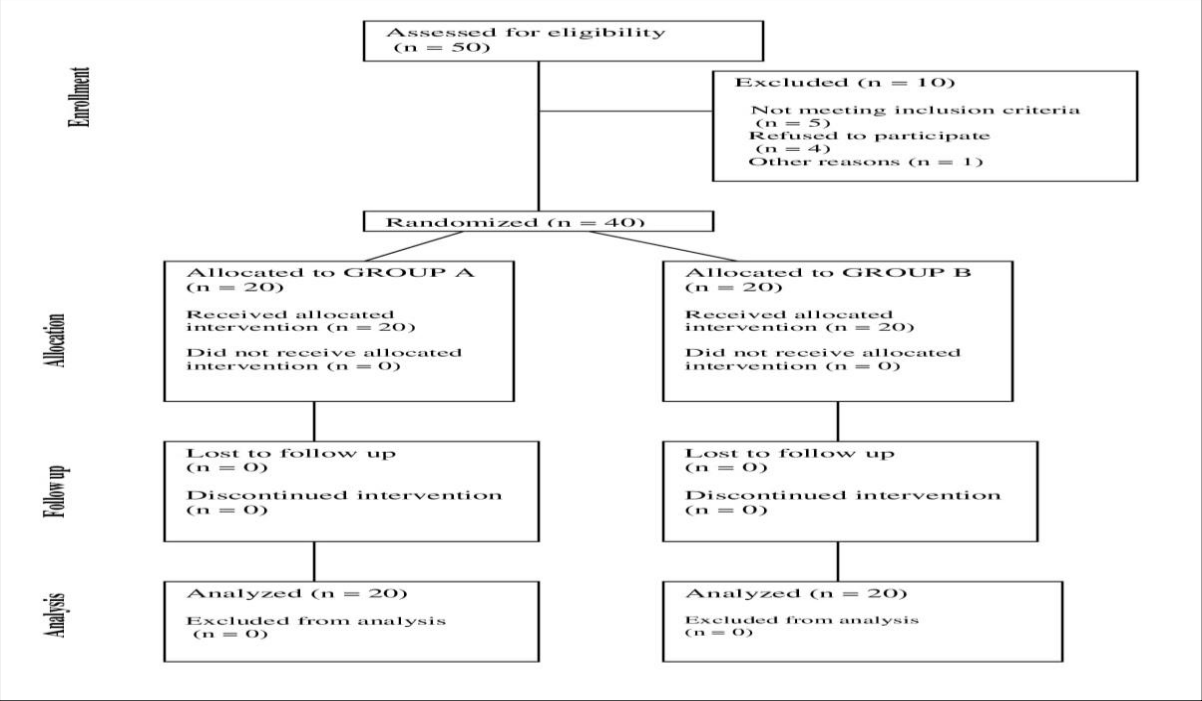
Consort Flow Chart

Upon ethical committee approval (21/2016), this prospective randomized interventional study was conducted over a duration of 12 months from July 2016 to June 2017 with a sample size of 40 patients, and the sample size was based on the study by Businco et al. (6). The flow of participants is shown in Fig 1. 

Patients aged over 5 years and under 15 years with characteristic symptoms such as mouth breathing and snoring were included in the study after ensuring that they did not have any tonsil-related complaints and tonsillar enlargement was less than 25%. These patients underwent a soft-tissue X-ray of nasopharynx in the lateral view with their mouths open and endoscopic assessment of adenoid hypertrophy. The Clemens and McMurray scale helped to grade adenoid enlargement, as follows: Grade I - adenoid tissue occupying 1/3 of the vertical height of the choana; Grade II - up to 2/3; Grade III - from 2/3 to nearly all but not complete choanal obstruction; Grade IV - complete choanal obstruction. The selected patients were categorized by systematic random sampling into two groups (A and B), with 20 patients in each group. Group A patients underwent conventional curettage adenoidectomy, and Group B patients underwent endoscopy-assisted coblation adenoidectomy. Syndromic children, children with a previous history of adenoidectomy, history of bleeding disorders, history of congenital heart diseases, and oromaxillofacial anomalies were excluded from the study.

*Surgical Technique: *Conventional curettage adenoidectomy was performed with the patient positioned in Sister Rose’s position using St.Clair Thompson adenoid curette. With the patient in supine position, coblation adenoidectomy was performed using the coblation wand. During the procedure, a pediatric 0-degree endoscope was used for visualization, allowing the coblation wand to be navigated behind the soft palate toward the nasopharynx through the oral cavity. The power level in the coblation unit was set at 3 for coagulation and 7 for ablation of adenoids. Subsequently, the two groups were compared based on the following factors:

### Completeness of the removal

A diagnostic nasal endoscopy was performed 1 month after the surgery by comparing the preoperative and postoperative adenoid grades. Nasal endoscopy showing adenoid tissue less than 1/3 of the choanal height in the vertical dimension, was taken as complete tissue removal.

### Surgical blood loss

The calculation of intraoperative surgical blood loss was based on the number of gauze pieces that were used to pack the nasopharynx to achieve hemostasis. A fully soaked gauze piece was assigned a blood loss of 10 mL, while the gauze piece represented a blood loss of 5 mL when it was partially soaked. To calculate the intraoperative blood loss, the amount of blood collected in the suction apparatus was added to the blood loss estimated using the gauze pieces after subtracting the quantity of irrigating fluid used, especially in the coblation method.

### Operative duration

The time duration calculated from mouth gag application until the achievement of adequate hemostasis was considered as the duration of surgery.

**Fig 2:**
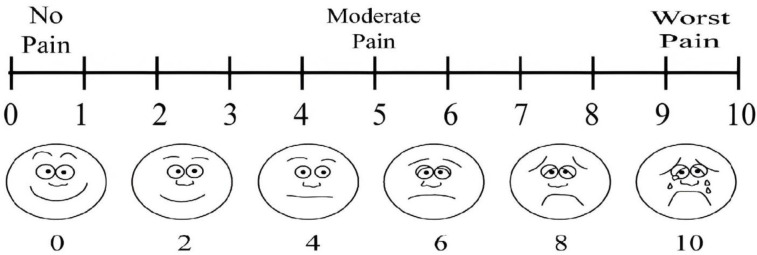
Visual analog scale (Image obtained from source (7))

**Fig 3:**
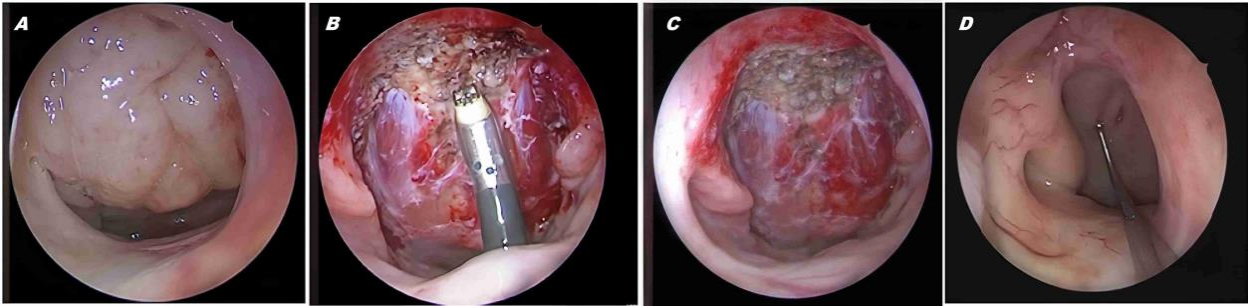
Perioperative images of adenoid. A: Grade 3 adenoid hypertrophy according to Clemens and McMurray scale, B: Intraoperative image of endoscopic coblation adenoidectomy, C:

*Postoperative pain and recovery time: *The Visual Analog Pain Scale (VAS) was used to assess postoperative pain score and recovery time. The pain score on the day of surgery was considered for assessment of postoperative pain. The day when the VAS score was less than 2 without any need for analgesics was considered as the recovery time. The VAS scale is shown in Figure 2 (7), and perioperative images of the procedure are depicted in Figure 3. 

IBM SPSS Statistics for Windows, Version 27 (IBM Corporation, New York, United States, 2021) was used for statistical analysis and associations were considered to be significant when the p-value was less than 0.05. Mean and standard deviation were used for continuous variables. Quantitative variables were expressed in terms of percentage and proportions. The comparison of different variables, such as gender, was analyzed using the chi-square test, while the parameter (completeness of removal) was analyzed using the Fischer’s exact test. For all other parameters using mean and standard deviation, Student’s T test for continuous variables was used.

## Results

 Among the 40 patients included in the study, conventional curettage adenoidectomy was performed in 20 patients of group A and endoscopy-assisted coblation adenoidectomy was performed in 20 patients of group B. Both the groups had comparable demographic characteristics. The mean age was 10.34 ± 3.18 in Group A and 9.68 ± 1.94 in Group B.

### Preoperative adenoid grading

Adenoid hypertrophy was assessed using the McMurray and Clemens scale under endoscopic visualization. Preoperatively, in Group A, 75% (n = 15) and in Group B, 70% (n = 14) had adenoid hypertrophy of grade 2. The mean grading was 2 ± 0.45 in Group A and 2 ± 0.55 in Group B (p = 0.126). The data regarding mean age and mean adenoid grading are summarized in Table 1. 

**Table 1:** Demographic characteristics and preoperative adenoid grade

	***Group A***	***Group B***	***p-value***
Age distribution*	10.34 ± 3.18	9.68 ± 1.94	0.088
*Sex distribution#*			p > 0.05
*Males n (%)*	12 (60%)	9 (45%)	
*Females n (%)*	8 (40%)	11 (65%)	
Preoperative grading of adenoids*	2 ± 0.45	2 ± 0.55	0.126

The data has been presented as n (%). * Data is expressed as mean± SD (standard deviation)

*Surgical blood loss:* In the coblation group, 10% (n = 2) of patients had less than 5mL blood loss, while none in the conventional group had less than 10 mL. The mean blood loss was 30 ± 5.60 and 10.75 ± 2.93 in Groups A and B (p = 0.0001) and is described in Table 2 .

**Table 2:** Surgical blood loss

Blood loss (in mL) *	***Group A n (%)***	***Group B n (%)***
*≤5*	0	2 (10%)
*5–10*	0	13 (65%)
*11–20*	1 (5%)	5 (25%)
*21–30*	10 (50%)	0
*31–40*	9 (45%)	0

* The data has been presented as n (%)

*Surgery* *duration:* The operative duration was less than 10 minutes in 65% (n = 13) of patients in the conventional group. In contrast, only 5% (n = 1) of patients had a duration under 10 minutes, while 20% (n = 4) had a operative duration up to 20 minutes in the coblation group. The mean surgical duration was 10.3 ± 3.33 minutes in Group A and 14.7 ± 2.34 minutes in Group B (p = 0.0001) and is described in Table 3.

**Table 3:** Duration of surgery

Duration in minutes*	**Group A n (%)**	**Group B n (%)**
<5	0	0
5–10	13 (65%)	1 (5%)
11–15	7 (35%)	15 (75%)
16–20	0	4 (20%)

* The data has been presented as n (%).

*Completeness of the removal: *Postoperative endoscopy with adenoid remnant less than 1/3 of the height of the choana in the vertical dimension was considered complete removal. According to this criterion, 15% (n = 3) of Group A patients and 75% (n = 15) of Group B patients showed complete removal. This showed a notable difference in the completeness of removal between both the groups (p = 0.0003) and is described in Table 4.

**Table 4:** Completeness of tissue removal

	**Group A n(%)**	**Group B n(%)**
Complete adenoid tissue removal	3 (15%)	15 (75%)
Partial removal	17 (85%)	5 (25%)

* The data has been presented as n (%).

*Postoperative pain:* On the day of surgery, the pain score was 4 in 80% (n = 16) of patients in Group A, while the pain score in 85% (n = 17) of patients in Group B was 3. The VAS score was 5 in 5%(n=1) patients on the day of surgery in Group A, whereas the highest VAS score in the coblation group was only 4. The median VAS score was 4 ± 0.44 in Group A and 3 ± 0.36 in Group B (p = 0.0001). The mean time taken for recovery was 3.14 ± 0.62 days in Group A and 2.64 ± 0.64 days in Group B (P = 0.001). Mean postoperative pain score, operative duration, and recovery period are described in table 5 and table 6.

**Table 5:** Postoperative VAS score

Pain score (POD–0*)	**Group A n(%)**	**Group B n(%)**
VAS scale score 1	0	0
VAS scale score 2	0	0
VAS scale score 3	3 (15%)	17 (85%)
VAS scale score 4	16 (80%)	3 (15%)
VAS scale score 5	1 (5%)	0

* The data has been presented as N (%). POD– postoperative day

**Table 6:** Mean postoperative pain score, operative duration, and recovery period

**Group A** **(mean ± Standard deviation)**	**Group B** **(mean ± Standard deviation)**	**p-value**	**Group A** **(mean ± Standard deviation)**
POD-0 Pain score*	4 ± 0.44	3 ±0.36	0.0001
Operative duration (in minutes) *	10.4 ± 3.23	14.6 ± 2.33	0.0001
Time for Recovery* (in days)	3.14± 0.62	2.64 ± 0.64	0.0017

*Data are expressed as mean ± SD (standard deviation)**;** POD – postoperative day

*Recovery time:* The recovery time was defined as the day when the postoperative VAS scale score was less than 2, thereby indicating the non-requirement of analgesics. 

Patients who underwent coblation adenoidectomy recovered earlier than those who underwent conventional adenoidectomy, as described in Table 7. 

**Table 7:** Day of recovery

Postoperative day on which VAS score <2*	**Group A n (%)**	**Group B n (%)**
Pod-1	0	8 (40%)
Pod-2	2 (10%)	12 (60%)
Pod-3	12 (60%)	0
Pod-4	6 (30%)	0

* The data has been presented as n (%); Pod – postoperative day

## Discussion

Adenoidectomy is a routine surgical procedure done in pediatric patients. Various techniques have been developed to reduce surgical blood loss and facilitate complete adenoid tissue removal. Simple adenoid curettage, adenoidectomy using bipolar cautery, powered microdebrider, and coblation-assisted adenoid removal are the current options for adenoid surgery (4,6). Choosing an appropriate technique is based on the efficacy of various techniques and their postoperative outcomes. The anatomical location of the adenoids can cause difficulties in accessing them, and the practice of conventional blind curettage methods has resulted in high recurrence rates of adenoid tissue (8,9). Endoscopic adenoidectomy enables removal of adenoid tissue under visualization of the entire nasopharynx, which facilitates complete removal without injuring adjacent vital structures (10). Coblation is a non-thermal process that employs bipolar radiofrequency energy for soft-tissue dissolution. The plasma generated in this process can both cut the tissue and cause coagulation. Reduced surgical bleeding is the hallmark of this non-thermal dissolution technique. It offers the advantage of early recovery since the patient experiences less postoperative pain (11,^12^). In our study, the adenoid grade was endoscopically assessed post-surgery at the end of one month. At the time of assessment, 15% of patients in the conventional group, and 75% in the coblation adenoidectomy group exhibited complete adenoid removal, with a p-value of 0.0003. This observation agrees with the findings of Songu et al. (13), who calculated the adenoid divided by nasopharyngeal ratios in CT temporal bone. This study revealed a notable statistical difference, with a mean ratio of 0.41 in the curettage group and 0.30 in the endoscopic adenoid group. Elnashar et al. reported results similar to our study when comparing endoscopic adenoidectomy and curettage adenoidectomy. The volume of adenoid tissue removed via conventional curettage adenoidectomy was between 1 and 3.6 mL, with a mean value of 2.45 mL, while the volume of adenoid removed via conventional curettage was between 0 and 2.9 mL, with a mean value of 0.66 ± 0.56 mL (14). Hence, the blind method resulted in incomplete removal of adenoids postoperatively. To avoid recurrence due to residual adenoid following blind curettage, Havas et al. proposed a combined technique that involved preliminary removal of adenoid tissue using the adenoid curette, followed by removal of residual adenoid tissue using a microdebrider (15). Pagella et al. described the transoral and endonasal technique of adenoidectomy (16). Xiao et al. compared conventional adenoidectomy and coblation adenoidectomy in 54 children who were categorized into two groups. Their study showed that the coblation group had less intraoperative bleeding, increased operative time, less postoperative pain, and a shorter recovery time. These results matched our study findings, in which the mean and SD of the intraoperative blood loss were 30 ± 5.60 mL in the conventional group and 10.75 ± 2.93 mL in the coblation group (p = 0.0001). On analyzing the postoperative pain, the median VAS score was 4 ± 0.44 in the conventional group and 3 ± 0.36 in the coblation group (p = 0.0001) (17).

 The pros and cons of coblation-assisted surgical technique and the conventional curettage technique were compared by Tahan et al. Operative time, amount of blood lost intraoperatively, pain in the postoperative period, complications encountered postoperatively, and late recurrences were recorded. The findings showed reduced operative duration in patients undergoing conventional adenoidectomy. In contrast, reduced intraoperative blood loss, postoperative bleeding, and recurrent adenoid hypertrophy were observed in patients undergoing coblation adenoidectomy. The two groups, however, showed no significant difference in postoperative pain (18). Similar to this study, the mean operative duration was 10.4 ± 3.23 min in the conventional group and 14.6 ± 2.33 min in the coblation group (p = 0.0001) in our study. 

 In a meta-analysis that was performed by Yang et al., coblation adenoidectomy under endoscopic guidance and conventional adenoidectomy were compared. In this study, 331 subjects underwent coblation adenoidectomy and 251 subjects underwent conventional adenoidectomy. Endoscopy-assisted coblation adenoidectomy had a shorter operative duration, reduced surgical blood loss and fewer complications (19). Regarding recovery time in the postoperative period, a review by Benninger and colleagues compared conventional cold dissection and coblation adenotonsillectomy. The authors showed that the coblation technique was associated with milder postoperative pain and thus decreased the requirement for postoperative narcotic usage, enabling a quicker recovery (20).

These results were similar to our study, where the mean recovery time was 3.14 ± 0.62 days for the conventional method and 2.64 ± 0.64 days for the coblation method with a p value of 0.001. Moreover, none of the patients in the coblation group required analgesics after the second postoperative day. Ozkiris et al. analyzed curettage and coblation techniques in terms of the amount of surgical blood loss, the duration of surgery, and mucociliary clearance rates in nose (NMCR) determined in the perioperative period. They showed that the coblation group exhibited better NMCR values (21). Similar to the aforementioned studies, our study showed that the coblation technique under endoscopic visualization was significantly better than the curettage adenoidectomy method in terms of complete adenoid tissue removal (22,23). The blind curettage method has an inherent risk of injuring the adjacent anatomical structures, which can lead to partial or incomplete removal. In contrast, endoscopic visualization provides a clear view of the nasopharynx, making meticulous removal possible. Regarding intraoperative blood loss, the coblation technique is better than the conventional method (24,^25^). 

In the coblation technique, when an electric current is passed through the conducting fluid, the bonds at the molecular level in the tissue are broken down by the released plasma, resulting in molecular-level disintegration. This technique, therefore, causes minimal bleeding (26). The postoperative VAS score was lower in the coblation method, which led to early recovery. Although patients were evaluated endoscopically in the postoperative period, late recurrences were not studied in either group. 

Limitations of the study: 

Although the coblation method is superior to the conventional technique, the coblation technique does have limitations according to literature. Conventional adenoidectomy is a simple procedure with a shorter learning curve than the coblation technique, which requires considerable training (27) to gain expertise. In addition, setting up the coblation system and positioning the patient for endoscopic visualization are time-consuming tasks (28). In contrast, the conventional method can be performed quickly without any such special arrangements. Another limitation is the high cost incurred with the coblation system and the single-use wands. Notwithstanding these limitations, prioritizing the outcomes outweighs the drawbacks when the results are considered on a long-term basis.

## Conclusion

The endoscopy-assisted coblation method of adenoidectomy provides more effective removal of adenoid tissue compared to the conventional curettage method. Patients who underwent coblation adenoidectomy experienced reduced intraoperative blood loss and postoperative pain, ensuring a shorter postoperative recovery period. However, the coblation method requires more time to position the patient and organize the equipment, resulting in a longer operative duration than conventional adenoidectomy. 
